# Pyroptosis-related gene signature for predicting gastric cancer prognosis

**DOI:** 10.3389/fonc.2024.1336734

**Published:** 2024-03-20

**Authors:** Salem Saeed Saad Khamis, Jianhua Lu, Yongdong Yi, Shangrui Rao, Weijian Sun

**Affiliations:** ^1^ The Second Affiliated Hospital and Yuying Children's Hospital of Wenzhou Medical University, Wenzhou, Zhejiang, China; ^2^ Department of General Surgery, Wenzhou Medical University First Affiliated Hospital, Wenzhou, Zhejiang, China

**Keywords:** pyroptosis, gene signature, gastric cancer, prognosis, inflammation

## Abstract

Gastric cancer (GC) is a prevalent form of malignancy characterized by significant heterogeneity. The development of a specific prediction model is of utmost importance to improve therapy alternatives. The presence of H. pylori can elicit pyroptosis, a notable carcinogenic process. Furthermore, the administration of chemotherapeutic drugs is often employed as a therapeutic approach to addressing this condition. In the present investigation, it was observed that there were variations in the production of 17 pyroptosis-regulating proteins between stomach tissue with tumor development and GC cells. The predictive relevance of each gene associated with pyroptosis was assessed using the cohort from the cancer genome atlas (TCGA). The least absolute shrinkage and selection operator (LASSO) was utilized to enhance the outcomes of the regression approach. Patients with gastric cancer GC in the cohort from the TCGA were categorized into low-risk or high-risk groups based on their gene expression profiles. Patients with a low risk of gastric cancer had a higher likelihood of survival compared to persons classified as high risk (P<0.0001). A subset of patients diagnosed with GC from a Genes Expression Omnibus (GEO) cohort was stratified according to their overall survival (OS) duration. The statistical analysis revealed a higher significance level (P=0.0063) regarding OS time among low-risk individuals. The study revealed that the GC risk score emerged as a significant independent prognostic factor for OS in patients diagnosed with GC. The results of Gene Ontology (GO) and Kyoto Encyclopaedia of Genes and Genomes (KEGG) research revealed that genes associated with a high-risk group had significantly elevated levels of immune system-related activity. Furthermore, it was found that the state of immunity was diminished within this particular group. The relationship between the immune response to cancer and pyroptosis genes is highly interconnected, suggesting that these genes have the potential to serve as prognostic indicators for GC.

## Background

Gastric cancer ranks as the third leading cause of cancer-related mortalities globally (1). Approximately one million novel cases of GC are reported annually basis (2) The disease frequently only becomes apparent in its advanced stages when the Tumor microenvironment (TME), which contributes to its diversity has changed. The responses to treatments among GC patients vary significantly, resulting in a poor prognosis (3). Restoring GSDME expression in gastric cancer cells was shown to enhance their sensitivity to chemotherapy and promote tumor shrinkage. Therefore, GSDME is considered a promising therapeutic target in GC. Moreover, its participation in pyroptosis presents an opportunity for the development of novel therapeutic strategies that use the immunogenic cell death process to tackle this dissease (4). Pyroptosis, an atypical form of programmed cell death distinguished by cell inflammation and necrosis. However, skin bulge stem cells have been notably observed in mice with Gsdma3 mutations (5). Abnormal DNA methylation was linked to colorectal cancer in the genome-wide profiling investigation. The study used regression analysis to examine GSDME methylation and expression, finding that CpG methylation affects expression differently. The study also found CpGs that predict tumor and non-tumor tissue states. The data show that GSDME methylation analysis may be a promising colorectal cancer biomarker for clinical practice (6). Pyroptotic cells exhibit bubble-like extensions and undergo expansion, with electron microscopy revealing the creation of numerous vesicles before the cell membrane breaks down and releases cellular matter (7). Cardiolipin, a member of the gasdermin protein family, has structural domains and phosphatidylinositol. Its N-terminal activating domain becomes inactive upon attachment to the membrane and localization at cell membrane holes (8, 9). Members of the gasdermin family promote the formation of pores in the cell membrane, allowing cellular content to leak out and triggering a mild inflammatory response (10, 11). Considering the link between pyroptosis genes and the immune response to cancer, these genes could serve as prognostic indicators for GC.

Both infection and tumorigenesis have been associated with pyroptosis, involving gasdermin proteins, proinflammatory cytokines, and inflammatory vesicles (12). Studies have shown that transgenic mice are at higher susceptibility to colon cancer in comparison to their wild-type counterparts. Unlike apoptosis, which triggers a passive immune response (13). pyroptosis activates and releases signaling molecules and cytokines associated with danger. The objective of this study is to ascertain the specific genes implicated in pyroptosis and assess their expression levels and predictive significance in both healthy gastric tissues and GC tissues.

Additionally, we aim to investigate how pyroptosis relates to the immune microenvironment of tumors. Previous studies have shown that pyroptosis changes the immune environment around a tumor. For example, the population and functional engagement of CD8+ T lymphocytes lacking GSDMD are decreased (14). fatty acid adipocyte migration and triacylglycerol production. PolyclonC3a and C5a, immune-related complement proteins, are downregulated by ASP (15). Complement system effector C3a activates and survives T lymphocytes and stimulates angiogenesis, chemotaxis, mast cell degranulation, and macrophages. It neutralises the proinflammatory effects of C5a and produces pro- and anti-inflammatory responses. C3a controls leukocyte growth in adaptive immunity. Human C3a impacts B cell and monocyte IL-6 and TNF-α production, lowering polyclonal immune response via dose-dependent B cell molecule synthesis (16). C3aR signaling on CD28 and CD40L pathways, antigen-presenting cells, affects T cell proliferation and differentiation. Regulatory T cell synthesis is increased by dendritic cell C3aR loss, but TH1 cell creation and IL-10 expression rely on it (17). while an absence of active C3aR on dendritic cells upregulates the production of T cells. C3a counteracts C5a, leading to diminished inflammation. C3a also blocks neutrophil migration and degranulation. C3a anaphylatoxins contain C-terminal arginine. Serum carboxypeptidase B cleaves the arginine residue of C3a to create desArg, an acylation-stimulating protein (17). Recent research has emphasized the significance of pyroptosis in the anti-tumor function of natural killer (NK) cells (18). Despite its acknowledged role in tumor growth and chemotherapy, the precise impact of pyroptosis in gastric cancer GC has not been thoroughly examined, thus requiring additional research in the present study.

## Methods and processes

### Datasets

We utilized TCGA data (https://portal.gdc.cancer.gov/) which comprised (RNA-seq) results and related clinical features for 375 GC patients. The RNA-seq data are available through Xenabrowser (https://xenabrowser.net/datapages/). Additionally, we used the GEO database (GSE62254) for data derived from RNA-seq experiments and corroborated clinical data from other sources. The follow-up duration for the GSE62254 cohort spanned up to 6 years, whereas the TCGA cohort had a minimum follow-up duration of 2 years.

### Genes differentially expressed in pyroptosis

We gathered data on 17 pyroptosis-related genes and compared their expression between tumors and normal tissues in the TCGA dataset, which comprises 32 normal gastric samples. For comparison, “limma” software identified genes with a P 0.05 significance level after normalizing FPKM data. The levels of significance were indicated as follows: *P < 0.05; **P < 0.01; ***P < 0.001. PPI networks were built using STRING Version 11.0, an interactive tool for retrieving interacting genes, accessible at https://string-db.org/.

### Gene prediction model development and validation for pyroptosis

In these experiments, we examined if pyroptotic-related genes could predict survival outcomes. We carried out a Cox regression analysis on the TCGA cohort, setting a cut-off of 0.2 for excluding. The aforementioned procedure resulted in the discovery of seven genes associated with survival. We used the LASSO Cox regression model from the R package ‘glmnet’ to refine our gene selection and build the prognostic model. Even after removing six genes and their coefficients, we kept the penalty parameter (λ) below the lowest qualification. To assess the TCGA dataset’s expression information, we utilized the R language’s scale function. Then, we calculated risk by multiplying each gene’s coefficient by its expression level. After analyzing the median risk score, we classified the TCGA cohort as either low-risk or high-risk. Using the Kaplan-Meier method, we evaluated the median duration of OS for each subgroup. We conducted PCA using the ‘prcomp’ function from the ‘stats’ package in the R programming language. Our analysis specifically focused on a six-gene signature.

In addition, we used the R packages’survminer,’’ survival,’ and ‘time ROC’ to conduct a six-year ROC curve analysis. To corroborate our research, we referred to the GEO database and employed the GC cohort (GSE62254). Consistent with the TCGA cohort, each gene involved in pyroptosis has its expression level standardized using the scale’ command. Following that, the risk score was computed using the same methodology. Participants from the GSE62254 cohort the individuals comprising the GSE62254 cohort were classified into two discrete groups based on their level of risk: low-risk and high-risk. A comparison investigation was undertaken to validate the accuracy and efficacy of the gene model.

### Independent predictive risk analysis

The TCGA and GEO cohorts were used for an independent predictive risk study.

The present research included an investigation into the relationship between patients’ ages and phases, revealing a collective impact on the risk score. This study made use of a variety of regression techniques, including multivariate and univariate analyses.

### DEG study for improved understanding of low- and high-risk genetic associations

To augment our comprehension of the genetic connections present in both low- and high-risk groups, research was done on DEG. Patients from the TCGA cohort with GC were divided into “low risk” or “high risk. We then identified DEGs in each group using additional criteria, including a mean log2FC of 1 and an FDR of 0.05 or lower. The DEGs were analyzed for GO and KEGG using the ‘cluster profile’ software. To assess the scores of invading immune cells and investigate the activation of immune pathways, we used the ‘give’ method to perform single-sample gene set enrichment analysis (GSA).

### Statistical analysis

Here, Gene expression was measured in normal and gastric cancer GC tissues. The overall survival was compared among subgroups using the Kaplan-Meier analysis with a log-rank test to determine significance. We performed univariate and multivariate Cox regression studies to gauge the risk model’s predictive power. The Mann-Whitney test was used to assess the degree of immune cell infiltration and activation of the immune system in both cohorts. R (v4.0.2), a free and open-source statistical analysis program, was used for all studies.

## Results

**Figure 1:**
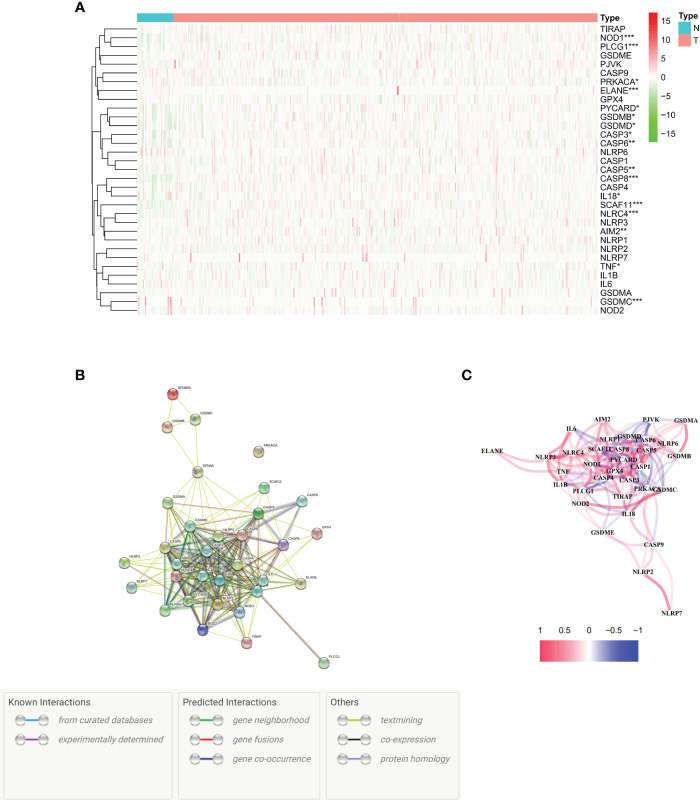
Expression and interactions of 33 pyroptosis-related genes. **(A)** Heat map illustrating the expression levels of pyroptosis-related genes in normal tissues (N, brilliant blue) and tumor tissues (T, red). The colors represent expression levels, with green indicating low expression and red indicating high expression (*P < 0.01; **P < 0.001; ***P < 0.0001). **(B)** Protein-Protein Interaction (PPI) network showcasing connections between pyroptosis-related genes with an interaction score of 0.9. **(C)**. Graph depicting the expression of genes positively (red line) and negatively (blue line) associated with pyroptosis. The intensity of the color reflects the strength of the association.

We conducted an analysis of differential gene expression in both normal and cancerous tissues. Seventeen genes associated with pyroptosis were identified in this study with significantly different expressions (P < 0.01) in the TCGA dataset, which comprises 375 tumors and 32 normal tissues. The genes showing decreased expression included NOD1, PLCG1, ELANE, IL18, SCAF11, NLRC4, AIM2, TNF, and GSDMC. On the other hand, there were more PRKACA, PYCARD, GSDMB, GSDMD, CASP3, CASP6, CASP5, and CASP8 in the tumor group (**Figure 1A**). To visualize these RNA levels, we created gene expression heatmaps, using green to indicate low expression and red for high expression. Our study of how proteins interact with each other showed that genes related to pyroptosis, such as GSDMC, NLRC4, SCAF11, CASP8, ELANE, PLCG1, and NOD1, are involved in many biological processes. The present investigation utilized a particular input parameter to ascertain the minimum permissible interaction score which we set at 0.9 for the PPI analysis (**Figure 1B**). This analysis included all genes associated with pyroptotic, forming a network of associations (**Figure 1C**). We denoted positive correlations with the color red and negative correlations with the color blue.

### Categorization of tumors using DEGs

**Figure 2:**
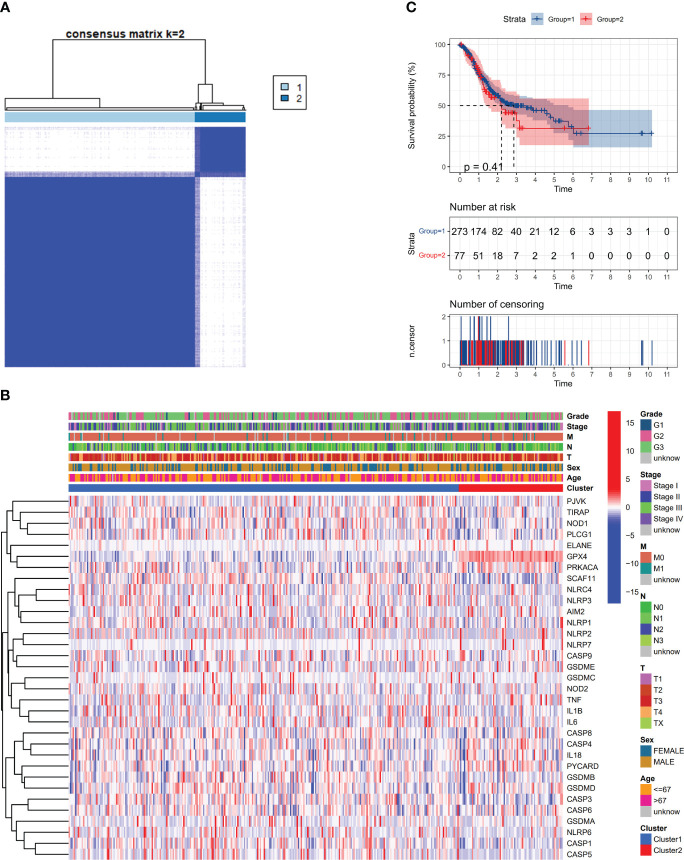
Apoptosis-related DEGs classify tumors. **(A)** An analysis classified 375 patients with gastric cancer (GC) into two groups using a consensus clustering matrix. **(B)** Heatmap of DEGs and clinicopathological characteristics of the clusters (G1, G2, and G3 represent tumor differentiation levels: G1 for highly differentiated, G2 for moderately differentiated, and G3 for poorly differentiated). **(C)** Kaplan-Meier plot illustrating overall survival (OS) development in the clusters.

This study examined the relationship between pyroptosis-related DEGs and gastric cancer varieties. analysis was conducted on a dataset comprising 375 members from the TCGA, utilizing consensus clustering for comparison. From the 31 identified DEGs, the 375 GC patients were effectively segregated into two unique groups (**Figure 2A**). A heatmap showed patient gene expression and clinical features. The variables incorporated in this study were tumor differentiation (graded as G1–G3), age (divided into either 67 years old or >67 years old), and the status of survival (categorized as being alive or dying). There were no statistically significant variations in clinical characteristics between the two groups (**Figure 2B**). The OS time showed no statistically significant difference between the two groups (P = 0.41) (**Figure 2C**).

### Gene prognostic model analysis of the TCGA cohort

We conducted an analysis of 375 genotyped gastric cancer samples, along with the corresponding patient survival data. The initial screening for genes associated with survival was carried out using univariate Cox regression analysis. A total of six genes, namely IL6, ELANE, GSDME, TIRAP, PYCARD, and CASP3, were selected for further investigation as they met the criterion of P < 0.01.

**Figure 3:**
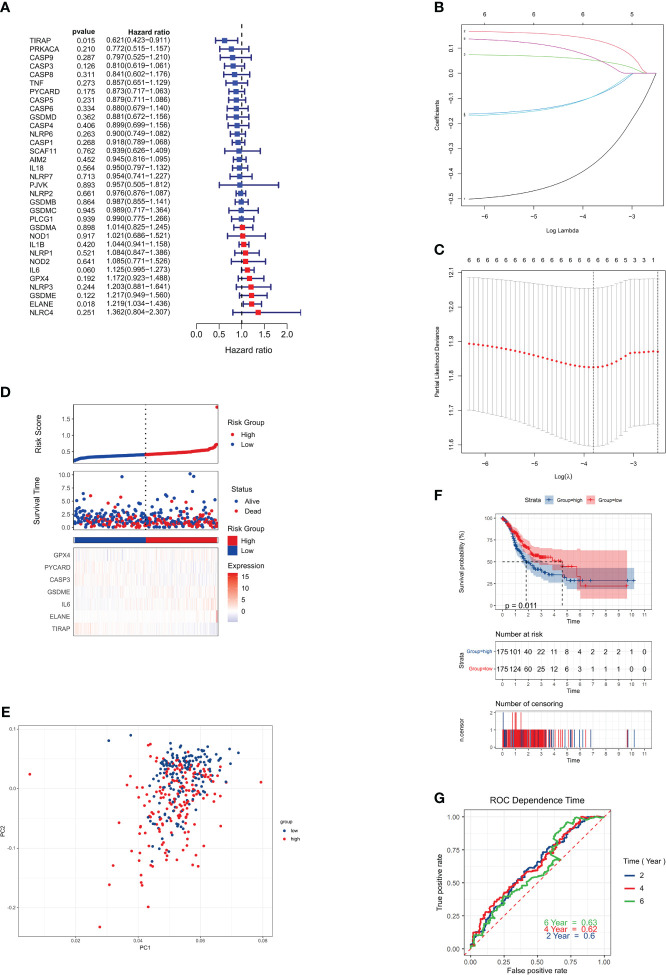
Risk signature for the TCGA cohort. **(A)** Univariate Cox regression analysis was performed on each pyroptosis-related gene and six other genes, with a P-value cutoff of 0.2. **(B)** LASSO regression was used to analyze the six genes associated with overall survival (OS). **(C)** LASSO regression parameters were adjusted through cross-validation. **(D)** Risk scores for the low-risk group are displayed on the left side of the dotted line, while those for the high-risk group are on the right side. **(E)** PCA plot for GCs based on the risk score. **(F)** Kaplan-Meier curves illustrate the risk categories and overall survival (OS) of patients (P = 0.0063). **(G)** ROC curve demonstrates the predictive ability of the risk score.

There was a significant association between IL6, ELANE, and GSDME and an increased chance of developing GC (HR > 1), while TIRAP, PYCARD, and CASP3 were shown to be associated with a reduced risk (HR < 1) (**Figure 3A**). To determine the best parameters with which to build a 6-gene signature (**Figures 3B, C**), we utilized LASSO Cox regression analysis. The risk score was computed via the subsequent mathematical expression: The expression may be written as follows: (0.060 multiplied by the exponential of IL6) + (0.018 multiplied by the exponential of ELANE) + (0.122 multiplied by the exponential of GSDME) + (0.015 multiplied by the exponential of TIRAP) + (0.175 multiplied by the exponential of PYCARD) + (-0.126 multiplied by the exponential of CASP3).

The 375 patients were classified into low-risk and high-risk categories, respectively, based on their median risk score (**Figure 3D**). The use of PCA. successfully classified patients with varying risk levels into these two categories (**Figure 3E**). Statistically significant disparities were seen in the overall survival durations between the low-risk and high-risk cohorts (P = 0.0063, **Figure 3F**). Finally, we used time-dependent ROC analysis to assess the prognostic model’s sensitivity and specificity. The ROC curves AUCs indicated 2-, 4-, and 6-year survival rates of 0.58, 0.61, and 0.63, respectively (**Figure 3G**).

### The risk signature’s independent verification

The risk signature was verified externally using the Genes and Expression Omnibus cohort, specifically applying the GSE62254 dataset. The ‘Scale’ tool was used to normalize the gene expression data before further investigation. Based on the median risk score of the TCGA cohort, the mean risk score was determined.

**Figure 4:**
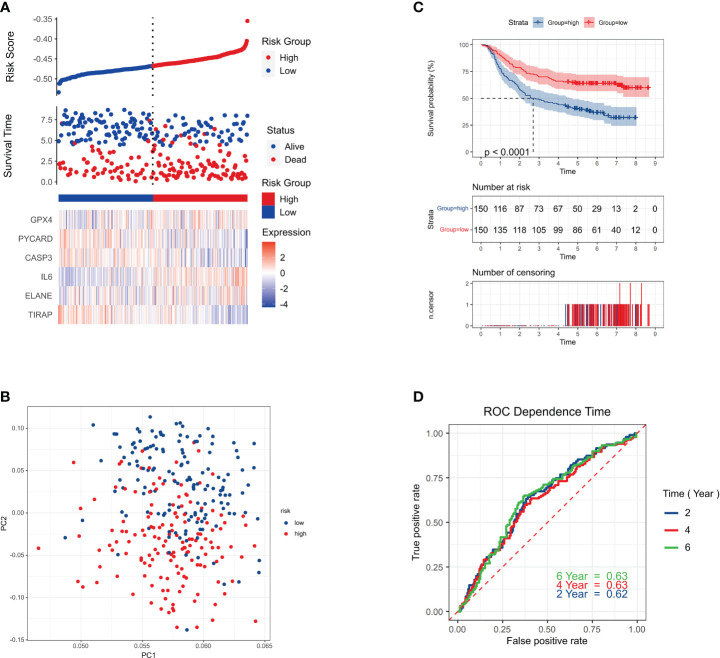
Survival status. **(A)** Survival status of each patient (low-risk population: left side of the dotted line; high-risk population: right side of the dotted line). **(B)** PCA plot of GC. **(C)** Kaplan–Meier curves for the comparison of the OS of low- and high-risk groups. P < 0.011. HR=1.63;95%CI(1.16-2.27) **(D)** Time-dependent ROC curves of GC.

Out of the individuals in the validation cohort, 273 were categorized as low risk while 77 surpassed the threshold, resulting in a high-risk classification. As seen by the solid line on the graph’s left, the low-risk category, demonstrated longer survival spans and decreased mortality rates compared to the high-risk group (**Figure 4A**). Principal Component Analysis showcased a distinct division between the two risk categories (**Figure 4B**). The low-risk and high-risk groups had significantly different survival rates (P = 0.011, HR = 1.63; 95% CI (1.16-2.27) (**Figure 4C**). AUC values of 0.62, 0.63, and 0.63 for 2-year, 4-year, and 6-year survival indicated strong prediction accuracy for the risk signature (**Figure 4D**).

### Risk model indicates independent prognostic value

**Figure 5:**
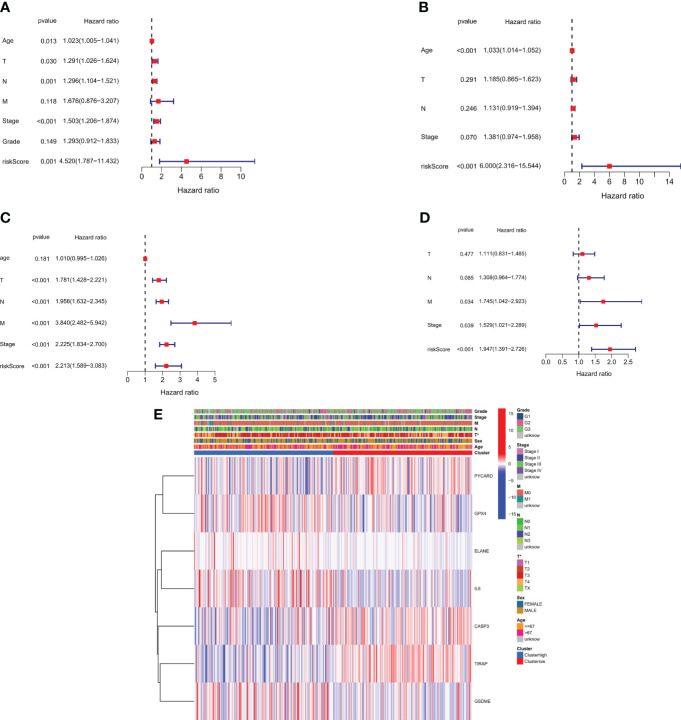
Risk score for univariate and multivariate Cox regression. **(A)** Univariate analysis of the TCGA cohort (tumor differentiation grade stages: G1 to G3). **(B)** Multivariate analysis of the TCGA cohort. **(C)** Univariate analysis of the GEO cohort (FIGO stage: I to IV). **(D)** Multivariate analysis of the GEO cohort. **(E)** Heatmap (green: low expression; red: high expression) showing the connections between clinicopathological features and risk grouping (*P < 0.05).

We performed univariate and multivariate Cox regression analyses to determine the utility of the risk score generated by the gene signature model. The independent variable in the univariate Cox regression analysis was the recognized risk score. Low survival rates were expected in the TCGA and GEO cohorts (HR = 4.520, 95% CI: 1.7873-11.432 and HR: 6.000, 95% CI: 2.316-15.544, (**Figures 5A, B**). Our multivariate analysis demonstrated that the risk score has prognostic predictive value after controlling for other confounding factors (HR = 2.213, 95% CI: 1.589-3.083 and HR = 1.947, 95% CI: 1.391-2.726, (**Figures 5C, D**). for patients diagnosed with gastric cancer in both study groups. Additionally, A heatmap was developed to visually represent the clinical features of the TCGA cohort, as shown in (**Figure 5E**). Notable differences in age and survival status were apparent between patients categorized in the low- and high-risk groups (P < 0.05).

### Functional risk assessments

**Figure 6:**
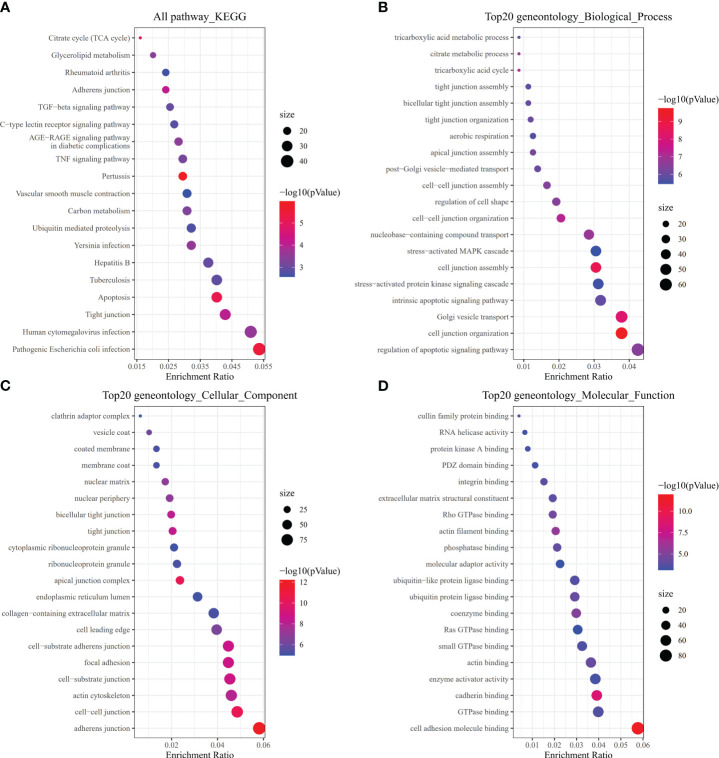
Pathway, biological, and molecular function analyses. **(A)** All KEGG pathways. **(B)** Top 20 GO biological processes. **(C)** Top 20 GO cellular components. **(D)** Top 20 GO molecular functions.

To investigate how risk model subgroups might differ in gene function and pathway, we identified DEGs using the ‘limma’ package in R, setting the FDR at 0.05 and the log2FC at 1. We performed GO enrichment analysis and KEGG pathway analysis based on the identified DEGs. Our research revealed that these DEGs primarily contribute to immune system signaling pathways, inflammatory cells, and chemokines (**Figures 6A–D**).

### Levels of immune activity in different subgroups

**Figure 7:**
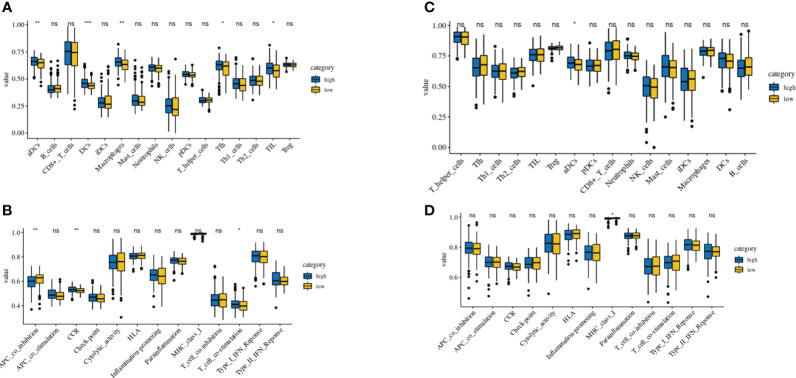
Comparison of the ssGSEA scores of immune cells and immune pathways. **(A, B)** Low-risk participants in the TCGA cohort had greater enrichment scores than those in the high-risk group based on the analysis of the 16 types of immune cells and 13 immune-related pathways. **(C, D)** Tumor immunity of the low-risk group (blue box) compared with that of the high-risk group (yellow box) in the GEO cohort. Results are represented by P values indicating that the data are nonsignificant; *P < 0.05; **P < 0.01; ***P < 0.001.

Using the TCGA and GEO cohorts, Using the ssGSEA, we analyzed the variance in array scores for immune cell**s (** 19–23). Thirteen immune-related pathways were activated in the low-risk and high-risk TCGA cohort groups, as shown by the ssGSEA technique (**Figure 7A**). High-risk individuals had fewer infiltrating immune cells such as CD8+ T cells, neutrophils, (NK) cells, and T helper cells (Tfh, Th1, and Th2 cells), In addition, the analysis of the TCGA cohort demonstrated that the high-risk group had decreased activity levels in 12 immune pathways, except the type-2 IFN response pathway, in comparison to the low-risk group (**Figure 7B**). Similar findings were seen during the evaluation of the immunological state of the GEO cohort. Notably, the low-risk group showed an abundance of IDCs and macrophages, while the expression of type-2 interferon (IFN) responses was significantly reduced (**Figures 7C, D**).

## Discussion

The present investigation revealed substantial disparities in the levels of expression of 33 genes related to pyroptosis in GC cells compared to normal tissues. Nevertheless, the consensus clustering analysis conducted on the DEGs did not reveal any statistically significant distinctions in the clinical features between the two groups. To evaluate the predictive value of these findings, we created a set of six regulators consisting of pyroptosis-related regulating genes. We utilized LASSO Cox regression analysis to gather the necessary data for this study, and an external dataset was further subjected to Cox univariate analysis.

The results of the functional analyses revealed statistically significant disparities in DEGs linked to immune-related pathways when comparing the low-risk and high-risk groups. Despite comparable levels of immune cell infiltration, Immune system activation was much lower in the high-risk group than in the low-risk group. Pyroptosis, an exceptional kind of controlled cellular demise, has garnered recent attention due to its pivotal involvement in both neoplastic proliferation and therapeutic interventions. The initiation of this process is prompted by heightened concentrations of inflammatory chemicals, hence potentially resulting in the genesis of malignant cells (24). As a result, focusing on tumor cell pyroptosis might pave the way for novel cancer treatments (25). However, the relationship between genes related to pyroptosis and patient survival in GC is still ambiguous. In this study, we constructed a pyroptosis signature composed of six genes including IL6, ELANE, GSDME, TIRAP, PYCARD, and CASP3 to predict the overall survival rate of GC patients. IL-6, a kind of cytokine recognized for its ability to induce inflammatory responses, is synthesized by several cell types, such as lymphocytes and monocytes. Autoimmune disorders have been shown to correlate with heightened levels of IL-6 and its corresponding receptor (26). Research has indicated that IL-6 can foster the growth of T-helper type 17 cells (25).

Recent research has provided valuable insights into the prominent involvement of IL-6 in several physiological mechanisms. These include cell proliferation, programmed cell death apoptosis, EMT, invasion, and cell migration. Collectively, these actions contribute to the progression and evolution of cancer.

IL-6 plays a key role in numerous protein kinase signaling pathways, the specifics of which depend on the cell type involved. CAR-T treatment can cause toxicities such cytokine release syndrome (CRS) and neurotoxicity due to increased immunological activation and inflammatory cytokines such as IL-6 and IL-1β from monocytes/macrophages. Pyroptotic cells also release DAMPs, which activate macrophages and produce cytokines, activating endothelial cells and CARTOX. The results emphasized the role of monocyte/macrophage in CARTOX and the relevance of pyroptotic cell-generated DAMPs as key contributors and therapeutic targets (27). The effects of chemokines on T cell trafficking and tumor cell metastasis, TLR-DAMP interactions in macrophages and dendritic cells, and cancer treatment targeting the DAMP-TLR-cytokine axis are discussed (28).

The protein ELANE, sometimes referred to as eosinophil cationic protein, is a crucial serine protease involved in the synthesis of tumor necrosis factor-alpha, interleukin 1 beta, and interleukin 18 (29, 30). The extensively established activation of pyroptosis-inducing pathways by these cytokines underscores the significance of ELANE (31). The cleavage and activation of GSDMD by this protein triggers pyroptosis in neutrophils. Despite the high-risk group having a high neutrophil infiltration score, the low-risk group has a much greater expression of ELANE, potentially due to the role of ELANE in stimulating pyroptosis in neutrophils.

GSDME/DFNA5, a member of the gasdermin superfamily (32, 33), is specifically expressed in various tissues such as skin and gastrointestinal tract epithelium (34). GSDME has been shown to serve as a tumor suppressor gene in several cases of stomach, colorectal, and breast malignancies.

It undergoes cleavage and caspase-3 activation in response to both intrinsic and extrinsic apoptotic therapies, leading to pyroptosis, similar to the membrane perforation effects induced by GSDMD (35, 36).

However, the role of GSDME in GC cells has yet to be fully elucidated. GC cells have shown susceptibility to pyroptosis induced by gastrin E GSDME under certain treatment circumstances (37). The notable expression of GSDME could suggest its involvement in widespread pyroptosis (38).

The adapter protein known as MyD88 adapter-like (MAL), or TIRAP (38, 39), is associated with the activation of the host immune response through receptor-mediated mechanisms. It is involved in the innate immune system’s detection of microbial infections via toll-like receptors (40, 41). The PIP2-binding domain (PBD) of MAL allows it to interact with the plasma membrane. This process entails the translocation of MAL to distinct regions on the cellular membrane after the production of PIP2 mediated by PIP5Ka (42–45). The protein known as MAL is comprised of two discrete domains, namely the N-terminal PYD domain and the C-terminal CARD domain, Extrinsic and intrinsic cell death processes depend on these domains (46, 47). PYCARD, also known as ASC, regulates inflammatory and apoptotic signaling pathways. The PYCARD domain of the protein facilitates the assembly of inflammasome complexes by interacting with sensor proteins AIM2, NLRP3 and Caspase-1 (48).

The interaction between PYD and sensor proteins, as well as between ASC and caspase-1, involves the binding of CARD domains. The assembly of inflammasome complexes triggers neutrophil inflammation, resulting in the cleavage, maturation, and subsequent release of IL-1 via the activation of pro-caspase-1, ultimately inducing inflammation (38, 49). Caspase 3, a regulatory protein for cellular apoptosis, is typically dormant. However, the initiation of apoptosis occurs through the activation of Caspase-3, which leads to the cleavage of several structural and regulatory proteins in both the nuclear and cytoplasmic compartments, thus inducing apoptosis (50).

GSDME-N, preferentially cleaved by caspase 3, penetrates membranes, thereby triggering pyroptosis (51). Patients with longer survival periods have been found to have increased levels of Caspase 3, indicating it may have a function in increasing sensitivity to the pyroptosis generated by chemotherapeutic drugs. In addition to boosting NLRP3, ASC, and Caspase 1 inflammasome activation, Caspase 6 has been shown to induce GSDMD-induced pyroptosis (52).

In our model, TIRAP, Caspase 3, and PYCARD were identified as genes promoting pyroptosis, while IL6, GSDME, and ELANE were identified as genes executing pyroptosis. However, it is important to note that modulating these genes did not improve the condition of gastric cancer in our study. Further investigation is required to have a comprehensive understanding of the molecular interactions among these genes in the context of pyroptosis. Biological process GO (BP, MF, and CC) annotations are shown in **Figure 6**. while Figure D displays the KEGG pathway analysis of the 20 most significant GO enrichment keywords for immune-related DEGs between thoracic aortic aneurysm and dissection (TAAD) and normal tissues.

While pyroptosis shares considerable similarities with apoptosis, our understanding of the former remains limited. Throughout the progression of a tumor, various cell death mechanisms can concurrently exist. Our model includes TIRAP, Casp3, and PYCARD, all of which play regulatory roles in apoptotic pathways. Unlike apoptotic cells that maintain intact membranes and don’t trigger inflammatory responses, pyroptotic cells rupture their membranes and release their contents. The genes identified as differentially expressed primarily contribute to the chemotaxis of inflammatory cells and immune response, suggesting their possible role in triggering cell death.

Given the limited research on pyroptosis, our investigation primarily focused on its mechanism in GC. The three gastrin family genes identified, which potentially induce pyroptosis in GC, along with the other three regulatory genes, could also impact other disorders.

We performed an initial evaluation of these pyroptosis-related genes’ predictive value, aiming to lay the groundwork for future research. However, we have not yet confirmed the role of these regulators in the pyroptosis pathways in GC, underscoring the need for further investigation. Our research has shown a strong correlation between pyroptosis and GCAs is apparent from the observed variations in the expression of genes associated with pyroptosis between normal and gastric cancer tissues. The risk signature based on our six-gene pyroptosis-related panel accurately predicted OS across multiple trials, with significant results.

## Conclusions

We observed a notable correlation between tumor immunity and DEGs in both low- and high-risk groups. To investigate GC immunity and pyroptosis-related genes, we created a gene profile for people who might react well to therapy. Our theory suggests that pyroptosis influences tumor composition by triggering severe inflammatory responses within an antibody and phagocyte-dependent microenvironment. In the TCGA and GEO cohorts with high cancer recurrence risks, immune systems appeared compromised.

Our findings revealed a higher prevalence of Treg cells in the low-risk group than in the high-risk group. This discrepancy could be due to the regulatory effect of Treg cells on the inflammatory responses incited by pyroptosis within the TME. We identified Treg cells with divergent functions in TME regulation and found two main subtypes with contrasting regulatory activities. Therefore, insight into the various subtypes of Treg cells in GC is essential for understanding their role in modulating the TME. Moreover, immune pathways in the high-risk groups of both cohorts demonstrated reduced activity. In conclusion, patients with high-risk GCs are more likely to have a poor prognosis because of diminished antitumor immunity.

## Data availability statement

The macrophage and gastric datasets can be downloaded at https://www.ncbi.nlm.nih.gov/geo/ the accession number GSE62254 and the RNA-seq data are available at https://xenabrowser.net/datapages/.

## Author contributions

KS: Formal analysis, Investigation, Resources, Visualization, Writing – original draft, Writing – review & editing. JL: Investigation, Resources, Validation, Visualization, Writing – review & editing. YY: Writing – review & editing. SR: Project administration, Supervision, Writing – review & editing. WS: Conceptualization, Formal analysis, Funding acquisition, Investigation, Resources, Supervision, Validation, Visualization, Writing – review & editing.
