# Supplementary figures and images for: Pyroptosis-related gene signature for predicting gastric cancer prognosis
Source: Front Oncol. 2024 Mar 20;14:1336734. doi: 10.3389/fonc.2024.1336734 (PMC10990040; doi:10.3389/fonc.2024.1336734)

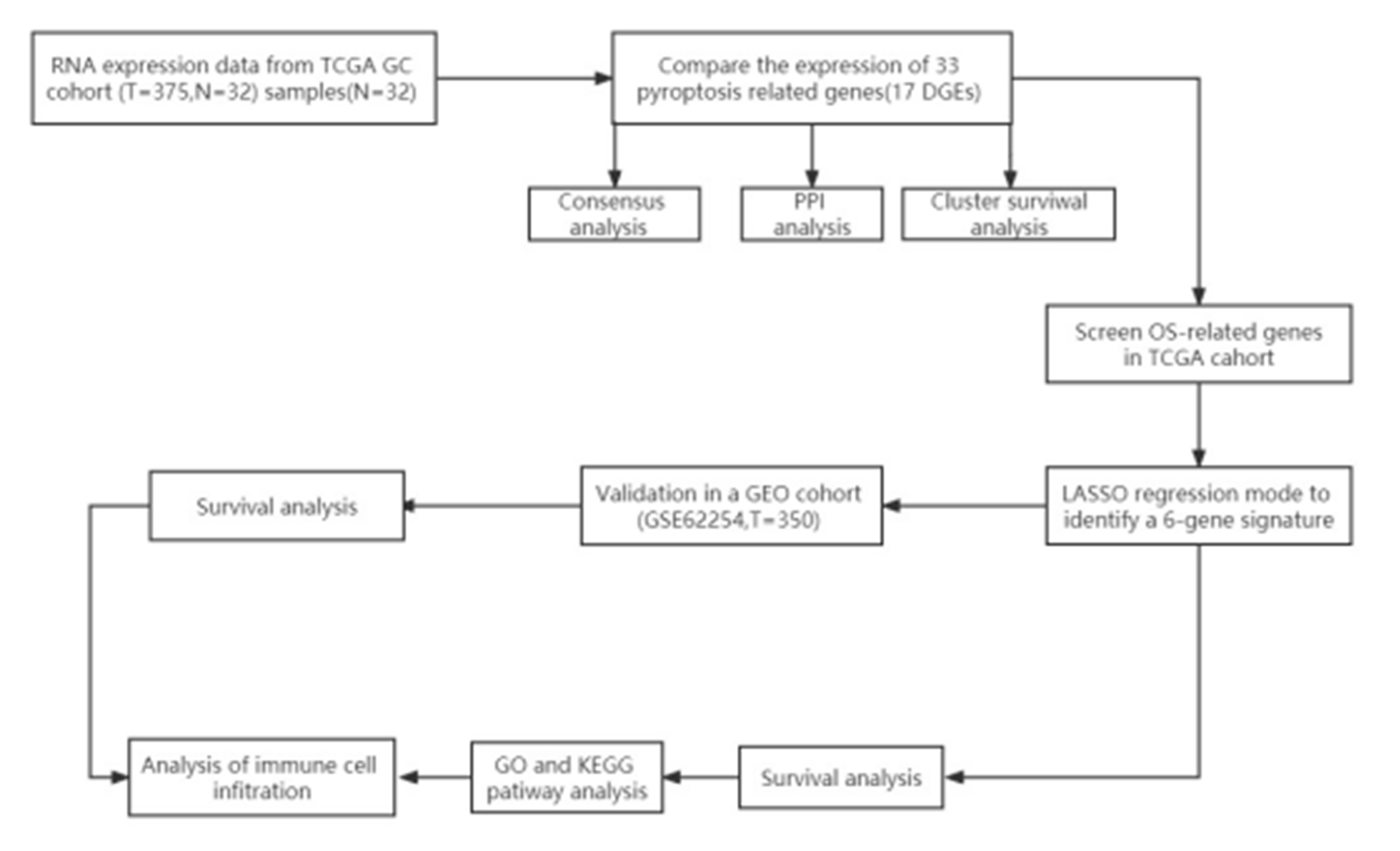

Supplement: Supplementary file 1 [file Image_1.tif]
